# Predictive Risk Factors and Online Nomograms for Synchronous Colon Cancer With Liver Metastasis

**DOI:** 10.3389/fonc.2020.01681

**Published:** 2020-10-02

**Authors:** Ya-Juan Zhu, Ye Chen, Hao-Yue Hu, Yu-Wen Zhou, Yue-Ting Zhu, Ji-Yan Liu

**Affiliations:** ^1^Department of Biotherapy, Cancer Center, and National Clinical Research Center for Geriatrics, West China Hospital, West China Medical School, Sichuan University, Chengdu, China; ^2^Department of Abdominal Cancer, Cancer Center, West China Hospital, West China Medical School, Sichuan University, Chengdu, China; ^3^Lung Cancer Center, Cancer Center, West China Hospital, West China Medical School, Sichuan University, Chengdu, China; ^4^Department of Oncology, The First People’s Hospital of Ziyang, Ziyang, China

**Keywords:** colon cancer, liver metastasis, SEER, prognosis, nomogram

## Abstract

**Objectives:**

To develop and validate predictive nomograms of cancer specific survival (CSS) and overall survival (OS) for synchronous colon cancer with liver metastasis (SCLM) patients.

**Methods:**

Patients with pathologically diagnosed colon cancer with liver metastasis were retrieved from the SEER database between 2010 and 2015. Only SCLM patients were included. Univariate and multivariate cox regression analyses were conducted to identify the potential predictors of patients’ survival outcomes. The selected variables were integrated to create predictive nomograms via R tools. Furthermore, the concordance index Harrell’s C statistic (C-index) was calculated to describe the discrimination of nomograms. Calibration (1000 bootstrap resamples) curves were plotted to compare the predictions of nomograms with the observed outcomes. Decision curve analysis (DCA) and clinical impact curves were performed to evaluate the clinical effects of nomograms.

**Results:**

A total of 22,378 SCLM patients were included. The median time of OS and CSS was 13 and 17 months, respectively. The 1-, 2-, and 3-year rate of OS was 50.6, 28.1, and 14.8%, respectively. While the 1-, 2-, and 3-year rate of CSS was 58.7, 36.8, and 22.5%, respectively. SCLM patients with increased age, left primary tumor location, AJCC IVb stage, and no chemotherapy were associated with an obviously reduced OS and CSS. Variables including age, histological grade, T/N/M stage, tumor size, bone/lung metastasis, CEA, surgery of primary site, and chemotherapy were closely related to the prognoses of SCLM patients. Nomograms of OS and CSS were built and displayed online for convenient utilization. The C-index of OS and CSS monograms were 0.74 and 0.73, respectively, indicating relatively good discrimination of the nomograms. The calibration curves suggested a good agreement between the actual observation and the nomogram prediction. DCAs and clinical impact curves reflected favorable potential clinical effects of predictive nomograms.

**Conclusion:**

Chemotherapy, surgery of primary site, and age were important independent risk factors for the CSS and OS of SCLM patients. We built and validated two reliable nomograms of OS and CSS to predict the prognoses of SCLM patients, which can be accessed online at (https://predictive-tool.shinyapps.io/CSS-DynNomapp/; https://predictive-tool.shinyapps.io/OS-DynNomapp/).

## Introduction

Colorectal cancer (CRC) is a commonly diagnosed malignant digestive tract cancer both in men and women worldwide. CRC is responsible for 10% of cancer-specific deaths in the United States, ranking as the second leading cause (1). Like other solid tumors, the distant metastasis is an essential prognostic factor of poor cancer survival. Most distantly metastatic CRC patients have only approximately a 13.5% chance of 5-year survival, while locally advanced patients have a favorable survival rate of 71% (2). Despite the difference in primary site and histology subtypes, generally, the most frequently metastatic organ of CRC is the liver, followed by the lungs, bone, and the brain (3). Specifically, liver metastases were observed in more than 25% of CRC patients when initially diagnosed. Liver metastases occurred in up to 25% of patients after the resection of a primary tumor. A total of 50% of CRC patients may develop liver metastases during the whole disease course (4, 5).

Of note, colon cancer patients have a higher metastatic potential for liver rather than rectal cancer. The most well-known mechanism is that the metastatic pattern is different due to the direction of hematogenous metastasis of colon cancer and rectal cancer. In colon cancer, the majority of the intestinal mesenteric drainage enters the hepatic portal venous system. Therefore, the liver is the primary organ involved. Whereas, the most common metastatic site of rectal cancer is the lungs since the rectum venous-collected blood flows into the systemic circulation (6).

The surveillance, epidemiology, and end results (SEER) database covers most of the cancer population from 18 American registries, thus providing opportunities to estimate the sociodemographic and clinical predictors of cancer prognosis in a large population (7). Nomograms are useful tools that can assist in quantitatively predicting the prognosis for each patient (8). Previous retrospective studies based on the SEER database has assessed the risk factors of poor survival for CRC patients with lung and bone metastasis and established a nomogram to estimate the cancer survival, respectively (9, 10). Synchronous colon cancer with liver metastasis (SCLM), a subtype of colon cancer with liver metastasis, is characterized with poor prognosis. The treatment for SCLM patients is also controversial. However, the patients’ characteristics and survival pattern of SCLM is still not clear.

In this study, we aimed to perform a retrospective analysis to investigate the pathological characteristics and treatment experience of SCLM patients using data from the SEER database. Furthermore, we intended to identify potential prognostic factors and build original predictive models for evaluating 1-, 3-, and 5-year cancer-specific survival (CSS) and overall survival (OS).

## Patients and Methods

### Study Populations

Based on the SEER database, patients diagnosed with primary colon cancer from 2010 to 2015 were retrospectively identified with the SEER^∗^Stat software version 8.3.6^1^. Patients with liver metastasis were selected. Individuals with the following information were excluded: unclear M stage, T0 stage, unclear survival time, or status of OS and CSS at the end of follow-ups. Variables including age, gender, race, histological grade, AJCC 7th TNM stage, tumor size, bone/lung/brain metastasis, CEA, surgery of primary site, surgery of liver metastasis, and chemotherapy were sorted. OS and CSS were defined as the primary outcomes. The follow-up time was defined as the time from diagnosis to death or to the last follow-up (December 31, 2015).

### Statistical Methods

The basic characteristics of the included patients were described with different variables. Univariate and multivariate cox analysis were performed to test each variable’s contribution in predicting survival outcomes and the hazard ratio (HR) was calculated with a corresponding 95% confidence interval (CI). Statistically significant risk factors were used to establish predictive nomograms of the 1-, 3-, and 5-year survival rate of individuals. The discrimination and calibration of nomograms were measured to evaluate the predicted probabilities of the nomogram. Calibration (1000 bootstrap resamples) curves were plotted to compare the predictions of the nomogram with observed outcomes. Decision curve analysis and clinical impact curves were performed to evaluate clinical effects of the nomogram (8, 11). The Kaplan-Meier method and groups were compared using the log-rank test when applicable. Statistical analyses were conducted via the SPSS version 25.0 (IBM Corporation, Armonk, NY, United States) and R software version 3.6.1^2^. *P*-value < 0.05 was considered as statistically significant.

## Results

### Patient Characteristics and Survival Outcomes

A total of 179,426 patients diagnosed with colon cancer were extracted from the SEER database. Of these, 22,697 patients were identified who had synchronous liver metastasis. After removing 253 patients with unavailable necessary information, 22,378 individuals were included and analyzed. The basic characteristics of the included patients are presented in Table 1.

**TABLE 1:** Baseline characteristics of colon cancer patients with synchronous liver metastasis.

Variables	Patients
N	22378
Median age (year)	66 ± 14
**Age**	
≤50	3097 (13.8%)
51–60	4658 (20.8%)
61–70	5666 (25.4%)
71–80	4742 (21.2%)
>80	4215 (18.8%)
**Gender**	
Female	10459 (46.7%)
Male	11919 (53.3%)
**Race**	
White	16760 (74.9%)
Black	3763 (16.8%)
Other	1855 (8.3%)
**Tumor primary site**	
Ascending colon	3680 (16.4%)
Transverse colon	1764 (7.9%)
Descending colon	1240 (5.5%)
Sigmoid colon	6394 (28.6%)
Other	9300 (41.6%)
**Grade**	
I	833 (3.7%)
II	10306 (46.1%)
III	3748 (16.7%)
IV	781 (3.5%)
Unknown	6710 (30%)
**AJCC stage**	
IVa	11631 (52%)
IVb	9312 (41.6%)
IVnos	1435 (6.4%)
**T**	
T1	2134 (9.5%)
T2	403 (1.8%)
T3	6818 (30.5%)
T4	5675 (25.4%)
Tx	7348 (32.8%)
**N**	
N0	7013 (31.3%)
N1	6835 (30.6%)
N2	5132 (22.9%)
Nx	3398 (15.2%)
**M**	
M1a	11639 (52.0%)
M1b	9306 (41.6%)
M1nos	1433 (6.4%)
**Surgery of primary site**	
No surgery	10933 (48.9%)
Tumor lesion	204 (0.9%)
Partial colectomy	4260 (19.0%)
Total/subtotal colectomy	6891 (30.8%)
Unknown	90 (0.4%)
**Surgery of liver metastasis**	
Yes	196 (0.9%)
No	22182 (99.1%)
**Bone metastasis**	
Yes	1157 (5.2%)
No	20460 (91.4%)
Unknown	761 (3.4%)
**Brain metastasis**	
Yes	209 (0.9%)
No	21336 (95.4%)
Unknown	833 (3.7%)
**Lung metastasis**	
Yes	4720 (21.1%)
No	16843 (75.3%)
Unknown	815 (3.6%)
**Tumor size**	
≤2 cm	370 (1.7%)
2–5 cm	7226 (32.3%)
5–10 cm	6108 (27.3%)
>10 cm	640 (2.8%)
Unknown	8034 (35.9%)
**CEA**	
Positive	12915 (57.7%)
Negative	1970 (8.8%)
Boardline	31 (0.2%)
Other	7462 (33.3%)
**Radiotherapy**	
Yes	754 (3.4%)
No	21624 (96.6%)
**Chemotherapy**	
Yes	13098 (58.5%)
No	9280 (41.5%)

*CEA, carcinoembryonic antigen; Grade I, well differentiated; Grade II, moderately differentiated; Grade III, poorly differentiated; Grade IV, undifferentiated.*

### Survival Outcomes

During the follow-up period, 64.8% (14500/22378) of patients died from SCLM. The survival outcomes showed that the 1-, 2-, and 3-year rate of OS was 50.6, 28.1, and 14.8%, respectively. The 1-, 2-, and 3-year rate of CSS was 58.7, 36.8, and 22.5%, respectively. The median time of OS and CSS was 13 months and 17 months, respectively.

**FIGURE 1:**
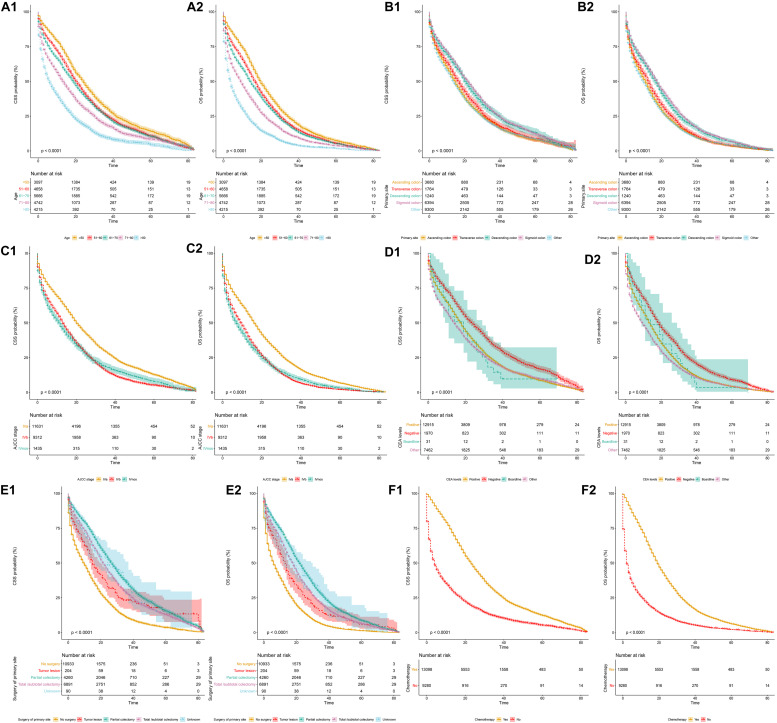
Cancer-specific and overall survival curves stratified by patient characteristics: **(A1,A2)** age; **(B1,B2)** primary tumor location; **(C1,C2)** AJCC stage; **(D1,D2)** CEA levels; **(E1,E2)** surgery of primary site; **(F1,F2)** chemotherapy.

When stratified by different variables, the OS and CSS of SCLM patients decreased significantly with an increase in age (Figures 1A1,A2). The survival outcomes of both OS and CSS were also influenced by different primary tumor locations. The prognosis of patients with a primary site of the ascending or transverse colon was significantly worse than those within the descending and sigmoid colon (Figures 1B1,B2). SCLM patients in the AJCC IVb stage were associated with obviously worse OS and CSS than those in IVa stage. The median OS of stage IVb patients was 9 months, while for stage IVa patients it was 16 months (Figures 1C1,C2). Patients with a negative CEA level had better CSS and OS prognosis than those with a positive CEA level [HR (95.0% CI) CSS 0.75 (0.70∼0.80), OS 0.75 (0.72∼0.80)] (Figures 1D1,D2). The SCLM patients that underwent partial colectomy and total/subtotal colectomy showed a relatively better prognosis that those without surgery (Figures 1E1,E2). The SCLM patients that received chemotherapy treatment had obviously better survival outcomes of both OS and CSS than those without chemotherapy (Figures 1F1,F2). The median CSS in SCLM patients with chemotherapy was 23 months compared to 4 months in patients without chemotherapy (*P* < 0.0001).

### Univariate and Multivariate Analyses

Variables that might possibly predict the CSS and OS of SCLM patients were analyzed. The results revealed that age, histological grade, T/N/M stage, tumor size, bone/lung metastasis, CEA, surgery of primary site, and chemotherapy were independent risk factors for the CSS and OS of SCLM patients. The factors of gender, brain metastasis, and radiotherapy seemed to have no significant relationship with the outcomes of OS and CSS. The detailed outcomes are presented in Table 2.

**TABLE 2:** Univariate and multivariatecox analyses of prognostic factors associated with CSS and OS in the studied cohort.

**Variables**	**CSS Univariate**		**CSS Multivariate**		**OS Univariate**		**OS Multivariate**	
	**HR (95.0% CI)**	***P* value**	**HR (95.0% CI)**	***P* value**	**HR (95.0% CI)**	***P* value**	**HR (95.0% CI)**	***P* value**
**Age at diagnosis, years**								
≤50	Reference		Reference		Reference		Reference	
51-60	1.18 (1.12∼1.25)	<0.01	1.11 (1.05∼1.18)	<0.01	1.17 (1.11∼1.23)	<0.01	1.10 (1.05∼1.16)	<0.01
61-70	1.27 (1.20∼1.34)	<0.01	1.15 (1.09∼1.22)	<0.01	1.27 (1.21∼1.33)	<0.01	1.15 (1.10∼1.21)	<0.01
71-80	1.61 (1.52∼1.70)	<0.01	1.35 (1.27∼1.43)	<0.01	1.63 (1.55∼1.71)	<0.01	1.36 (1.30∼1.43)	<0.01
>80	2.53 (2.38∼2.68)	<0.01	1.58 (1.48∼1.68)	<0.01	2.55 (2.42∼2.69)	<0.01	1.60 (1.51∼1.69)	<0.01
**Gender**								
Female****	Reference				Reference			
Male	0.98 (0.94∼1.00)	0.140	−	−	0.971 (0.94∼1.00)	0.060	−	−
**Race**								
White	Reference		Reference		Reference		Reference	
Black	0.98 (0.95∼1.03)	0.620	1.00 (0.95∼1.05)	0.950	1.01 (0.97∼1.05)	0.630	1.02 (0.98∼1.06)	0.280
**Tumor primary site**								
Ascending colon	Reference		Reference		Reference		Reference	
Transverse colon	0.92 (0.86∼0.99)	0.020	0.95 (0.88∼1.02)	0.140	0.93 (0.87∼0.99)	0.020	0.95 (0.90∼1.02)	0.141
Descending colon	0.77 (0.71∼0.84)	<0.01	0.89 (0.82∼0.96)	0.003	0.77 (0.72∼0.83)	<0.01	0.88 (0.82∼0.95)	<0.01
Sigmoid colon	0.74 (0.70∼0.77)	<0.01	0.81 (0.77∼0.86)	<0.01	0.73 (0.69∼0.76)	<0.01	0.80 (0.76∼0.84)	<0.01
**Grade**								
**I**	Reference		Reference		Reference		Reference	
II	0.99 (0.91∼1.08)	0.860	1.22 (1.11∼1.33)	<0.01	1.02 (0.94∼1.10)	0.711	1.25 (1.16∼1.35)	<0.01
III	1.40 (1.27∼1.53)	<0.01	1.61 (1.46∼1.77)	<0.01	1.44 (1.32∼1.56)	<0.01	1.67 (1.54∼1.82)	<0.01
IV	1.63 (1.45∼1.84)	<0.01	2.01 (1.78∼2.27)	<0.01	1.63 (1.47∼1.82)	<0.01	2.03 (1.82∼2.26)	<0.01
**T**								
T1	Reference		Reference		Reference		Reference	
T2	0.51 (0.45∼0.58)	<0.01	0.79 (0.69∼0.91)	0.001	0.51 (0.45∼0.57)	<0.01	0.78 (0.69∼0.89)	<0.01
T3	0.60 (0.57∼0.64)	<0.01	0.94 (0.87∼1.01)	0.094	0.60 (0.57∼0.64)	<0.01	0.94 (0.88∼1.00)	0.060
T4	0.80 (0.75∼0.85)	<0.01	1.08 (1.00∼1.17)	0.042	0.79 (0.75∼0.84)	<0.01	1.08 (1.09∼1.15)	0.029
**N**								
N0	Reference		Reference		Reference		Reference	
N1	0.85 (0.81∼0.88)	<0.01	1.09 (1.04∼1.14)	<0.01	0.84 (0.81∼0.87)	<0.01	1.07 (1.03∼1.12)	0.001
N2	0.85 (0.81∼0.89)	<0.01	1.35 (1.279∼1.43)	<0.01	0.84 (0.81∼0.88)	<0.01	1.33 (1.26∼1.39)	<0.01
**M**								
M1a	Reference		Reference		Reference		Reference	
M1b	1.47 (1.42∼1.52)	<0.01	1.22 (1.17∼1.28)	<0.01	1.48 (1.44∼1.53)	<0.01	1.23 (1.19∼1.28)	<0.01
**Bone metastasis**	****							
Yes	Reference		Reference		Reference		Reference	
No	0.57 (0.53∼0.62)	<0.01	0.78 (0.72∼0.84)	<0.01	0.58 (0.54∼0.61)	<0.01	0.79 (0.73∼0.84)	<0.01
**Tumor size**								
≤2 cm	Reference		Reference		Reference		Reference	
2–5 cm	1.12 (0.99∼1.28)	0.082	1.06 (0.93∼1.21)	0.385	1.14 (1.01∼1.28)	0.030	1.07 (0.95∼1.21)	0.244
5–10 cm	1.32 (1.16∼1.51)	<0.01	1.23 (1.071∼1.4)	0.003	1.29 (1.15∼1.45)	<0.01	1.20 (1.06∼1.35)	0.003
>10 cm	1.50 (1.28∼1.76)	<0.01	1.28 (1.091∼1.51)	0.003	1.45 (1.26∼1.67)	<0.01	1.24 (1.07∼1.43)	0.004
**CEA**								
Positive	Reference		Reference		Reference		Reference	
Negative	0.75 (0.70∼0.79)	<0.01	0.79 (0.74∼0.83)	<0.01	0.75 (0.72∼0.79)	<0.01	0.79 (0.75∼0.84)	<0.01
Boardline	1.08 (0.73∼1.60)	0.697	1.14 (0.77∼1.69)	0.510	0.99 (0.69∼1.43)	0.960	1.04 (0.72∼1.50)	0.823
**Brain metastasis**								
Yes	Reference		Reference		Reference		Reference	
No	0.58 (0.48∼0.69)	<0.01	0.89 (0.74∼1.06)	0.181	0.54 (0.46∼0.62)	<0.01	0.83 (0.71∼0.96)	0.055
**Lung metastasis**								
Yes	Reference		Reference		Reference		Reference	
No	0.71 (0.68∼0.74)	<0.01	0.95 (0.90∼0.99)	0.039	0.70 (0.67∼0.72)	<0.01	0.93 (0.89∼0.98)	<0.01
**Surgery of primary site**								
No surgery	Reference		Reference		Reference		Reference	
Tumor lesion	0.60 (0.50∼0.70)	<0.01	0.66 (0.55∼0.78)	<0.01	0.60 (0.51∼0.69)	<0.01	0.66 (0.57∼0.77)	<0.01
Partial colectomy	0.42 (0.40∼0.44)	<0.01	0.50 (0.47∼0.56)	<0.01	0.42 (0.41∼0.44)	<0.01	0.51 (0.48∼0.54)	<0.01
Total/subtotal colectomy	0.51 (0.49∼0.52)	<0.01	0.50 (0.47∼0.54)	<0.01	0.51 (0.49∼0.53)	<0.01	0.51 (0.48∼0.54)	<0.01
**Surgery of liver metastasis**								
Yes	Reference		Reference		Reference		Reference	
No	1.63 (1.37∼1.95)	<0.01	1.13 (0.95∼1.35)	0.181	1.55 (1.33∼1.81)	<0.01	1.08 (0.92∼1.26)	0.339
**Radiotherapy**	****							
Yes	Reference				Reference			
No	1.07 (0.97∼1.17)	0.165	−	−	1.06 (0.98∼1.15)	0.131	−	−
**Chemotherapy**	****							
Yes	Reference		Reference		Reference		Reference	
No	2.69 (2.60∼2.79)	<0.01	2.53 (2.44∼2.62)	<0.01	2.69 (2.61∼2.77)	<0.01	2.51 (2.42∼2.59)	<0.01

*CEA, carcino-embryonic antigen; Grade I, well differentiated; Grade II, moderately differentiated; Grade III, poorly differentiated; Grade IV, undifferentiated; CSS, cancer-specific survival; OS, overall survival; HR, hazard ratio; CI, confidence interval.*

### Nomograms and Calibrations

**FIGURE 2:**
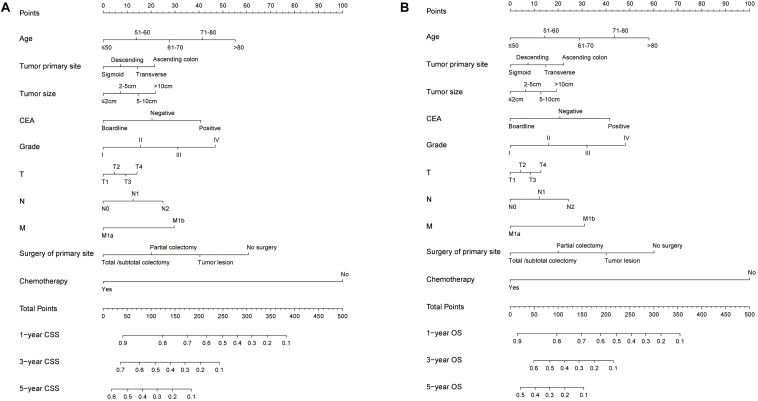
The predicting nomograms for the 1-, 3-, and 5-year CSS and OS of SCLM patients: **(A)** The nomogram for CSS; **(B)** The nomogram for OS.

**FIGURE 3:**
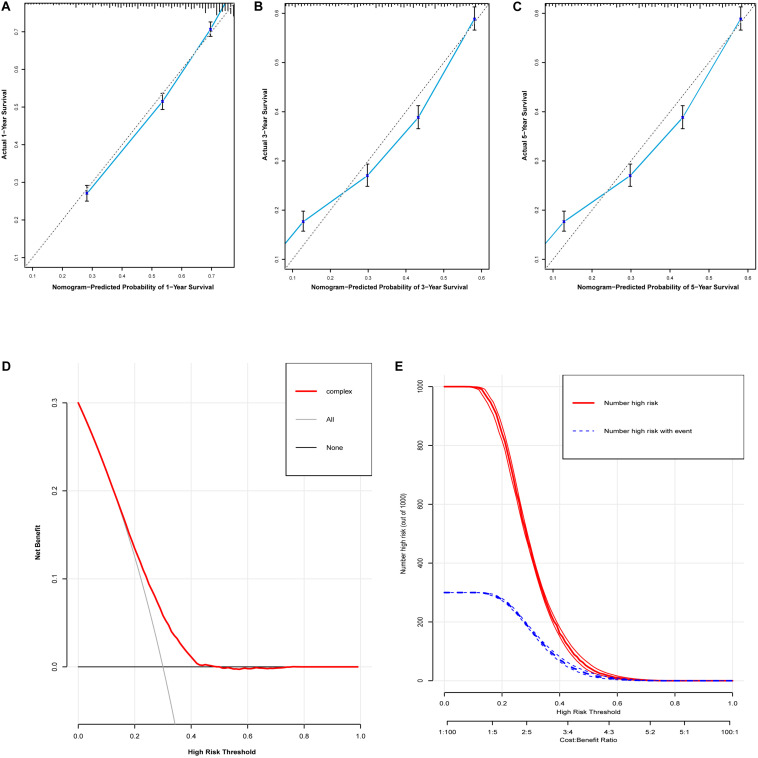
Evaluation of the nomogram for predicting CSS in the studied patients. **(A)** The calibration curves of 1-year CSS; **(B)** the calibration curves of 3-year CSS; **(C)** the calibration curves of 5-year CSS; **(D)** decision curves of CSS; **(E)** clinical impact curve of the predicted nomogram.

**FIGURE 4:**
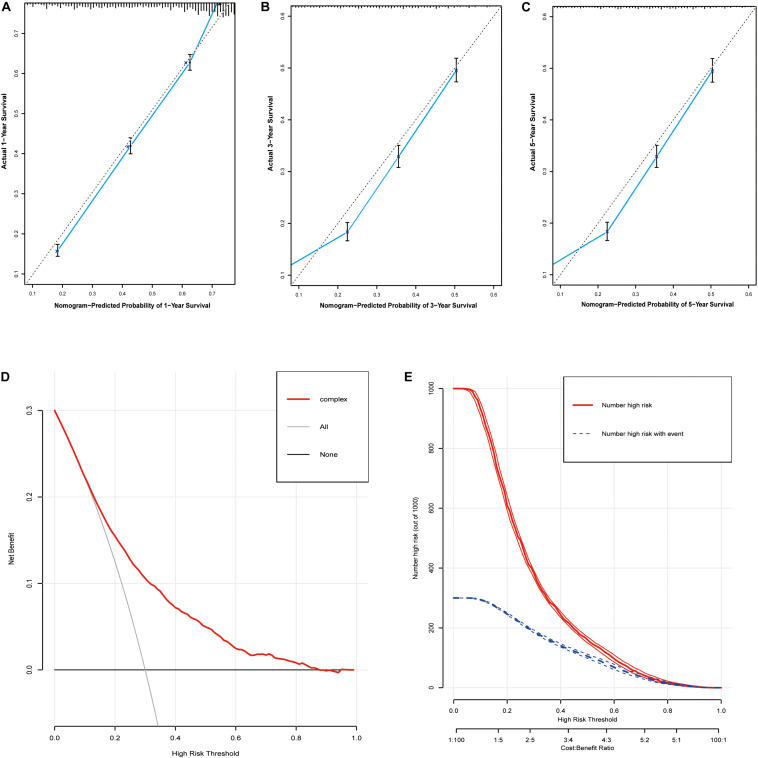
Evaluation of the nomogram for predicting OS in the studied patients. **(A)** The calibration curves of 1-year OS; **(B)** the calibration curves of 3-year OS; **(C)** the calibration curves of 5-year OS; **(D)** decision curves of OS; **(E)** clinical impact curve of the predicted nomogram.

Based on the predictive factors in the multivariable analysis, two nomograms were constructed to predict probabilities of CSS and OS (Figure 2). The C-index of two nomograms in predicting CSS and OS was 0.73 and 0.74, respectively, indicating good discrimination. The calibration curves of CSS and OS suggested a good agreement between the actual observed probabilities and predicted rates (Figures 3A–C, 4A–C). In addition, decision curve analysis (DCA) is a novel method to evaluate the net clinical benefit of a predictive model. DCAs reflected positive net benefits with a wide clinically reasonable risk threshold probability (Figures 3D, 4D). The clinical impact curves also represented acceptable potential clinical effects of the predictive nomograms (Figures 3E, 4E).

### The Webserver for Easy Access to Our Nomograms

We made an online version of our nomograms on the webserver^3^ ^,4^. After inputting the predictive variables on the webserver, the dynamic nomograms can easily display the calculated survival probabilities and generate relevant figures and tables.

## Discussion

Metastasis is closely related to the poor prognosis of patients with colon cancer. The liver is the most common organ of distant metastasis in advanced colon cancer (12). Based on the time of occurrence of liver metastasis, there are two types of synchronous and metachronous metastases of colon cancer. SCLM patients are commonly associated with obviously poorer prognoses. It is important to evaluate and predict the survival outcomes of SCLM patients. However, to the best of our knowledge, no nomogram has ever predicted the prognosis of SCLM patients. We extracted SCLM patients from the SEER database and built a predicting model of nomograms.

In our study, we included 22,378 SCLM patients. The low median time and survival rates of CSS and OS indicated that SCLM patients had poor prognoses in both OS and CSS. The lower median survival time is consistent with the reported overall survival time of synchronous colorectal cancer with liver metastasis patients (18.5 months) (13, 14). Similarly, another population-based study also revealed that the median overall survival time of SCLM patients is 7 months (15).

We analyzed the survival outcomes of included patients stratified by the factors of age, primary tumor site, AJCC stage, and chemotherapy. Our results found that the prognoses of SCLM patients were significantly reduced with the increase of age. Considering the primary tumor location of SCLM patients, SCLM patients with right-sided tumor location were associated with obviously poorer prognosis than those with other tumor sites. Similar results had been reported by some previous studies (13, 14, 16, 17). Compared with left-sided colorectal tumors, the liver metastatic area of right-sided tumors seemed to be more extensive, indicating that these patients had significantly worse prognoses (18). Our results indicated that SCLM patients with positive CEA levels had poorer prognoses than those with negative CEA levels. Previous studies have also demonstrated that CEA levels played an important role in the prognoses of SCLM patients (19–21). The SCLM patients with IVb of the AJCC stage showed obviously worse prognoses than those within the IVa stage. It suggested that SCLM patients combined with another distant organ or peritoneal metastasis had obviously poor prognoses. Other distant metastases, including bone and lung metastases, were important independent risk factors for the prognoses of SCLM patients. The metastasis of CRC to the brain is rare (3). Our results did not detect that brain metastases were significantly related with the prognosis of SCLM patients. It might be affected by the small number of patients with brain metastasis (209, 0.9%). The SCLM patients benefited from partial colectomy and total/subtotal colectomy comparing with those without surgery. Chemotherapy remarkably prolonged the survival time of SCLMs. In the clinical practice, chemotherapy and surgery are the most common effective treatments for SCLM patients due to significantly improved survival time of patients (22, 23).

In our study, the factors, including tumor primary site, tumor size, histological grade, T/N/M stage, surgery of primary site, and chemotherapy showed an association with the prognoses of SCLM patients. Our nomograms of both OS and CSS were built based on these factors. The C-index, calibration curves, and DCAs showed the excellent accuracy and consistency of the prediction models. In order to show the predicted results of our nomograms accurately, we established a user-friendly tool on an online webserver. The tool is available any time any place anywhere on mobile devices. It is convenient to detect the precise prognosis prediction for individual patients. A nomogram of a previous study also indicated that primary tumor location, lung metastasis, and CEA level were independent risk factors for the bone metastasis of colorectal patients (24). In another study, a nomogram was created to predict the probability of liver metastasis in patients with colon cancer (6). Some factors, including age, sex, race, tumor primary site, grade, and T/N stage were integrated in this nomogram. It calibrated well and had a high C-index (0.95). It could be an alternative to predict liver metastasis as a supplement to imaging tests.

There are some limitations in the present study. Firstly, even though we analyzed 22,378 patients, our study is still a retrospective study. There are some inevitable risks of bias and confounding factors in our study, which might influence the accuracy of our results. Secondly, we analyzed the patients from 2010 to 2015. However, the treatments for colon cancer and SCLM have been greatly updated in the past 5–10 years. New strategies, including percutaneous ablations, tumor embolization, and the introduction of new chemotherapeutic regimens as well as immune check point inhibitors are not mentioned. Therefore, the reference value of our results may be limited. High-quality studies with comprehensive and time-updated information are expected in the future. Thirdly, our nomograms are only tested by internal validations. Our results still need to be validated by data from the real world.

## Conclusion

In conclusion, the SCLM patients had poor prognoses. Variables including age, histological grade, T/N/M stage, tumor size, bone metastasis, lung metastasis, CEA, surgery of primary site, and chemotherapy were independent risk factors for SCLM patients. Nomograms of predicting the prognoses of SCLM patients were established and made available online. The nomograms were validated to be reliable and accurate for predicting the 1-, 3-, and 5-year OS and CSS rates of SCLM patients.

## Data Availability Statement

Publicly available datasets were analyzed in this study. This data can be found here: Surveillance, Epidemiology, and End Results (SEER) database (https://seer.cancer.gov/).

## Author Contributions

Y-JZ collected the data, performed the statistical analysis, and drafted the manuscript. YC substantially revised the manuscript and gave some meaningful suggestions on the modification. H-YH re-revised the manuscript. Y-WZ and Y-TZ supervised the data collection and analysis. J-YL designed the main study. All authors have read and approved the final submitted manuscript.

## Conflict of Interest

The authors declare that the research was conducted in the absence of any commercial or financial relationships that could be construed as a potential conflict of interest.

## Footnotes

1 www.seer.cancer.gov/seerstat

2 http://www.r-project.org

3 https://predictive-tool.shinyapps.io/CSS-DynNomapp/

4 https://predictive-tool.shinyapps.io/OS-DynNomapp/
